# The landscape of data sources for marine harmful algal bloom-related disease surveillance in the Philippines, Malaysia and Indonesia: a systematic scoping review

**DOI:** 10.14324/111.444/ucloe.3524

**Published:** 2026-10-08

**Authors:** Alisha Morsella, Lota Alcantara Creencia, Radisti A. Praptiwi, Nicolò Scarsi, Gaia Surya Lombardi, Walter Ricciardi, Lora E. Fleming, Chiara Cadeddu, Matthew O. Gribble

**Affiliations:** 1 Università Cattolica del Sacro Cuore, Rome, Italy; 2 University of Porto, Portugal; 3 Western Philippines University, Puerto Princesa, Philippines; 4 Research Center for Ecology, National Research and Innovation Agency (BRIN), Cibinong, Indonesia; 5 Institute for Biomedical Research and Innovation (CNR-IRIB), National Research Council of Italy, Istituto Marino Di Mortelle, Messina, Italy; 6 University of Perugia, Italy; 7 University of Exeter, UK; 8 Erasmus School of Health Policy & Management, Erasmus University Rotterdam, The Netherlands; 9 University of California San Francisco (UCSF), USA

**Keywords:** harmful algal bloom, Southeastern Asia, seafood, food safety, public health surveillance, data collection systems, environmental medicine

## Abstract

Harmful algal blooms occurring in the marine environment cause a variety of human illnesses in Southeast Asians, who rely on the marine ecosystem for sustenance, yet there is limited public understanding of these disease risks. Due to a lack of awareness, diagnostic uncertainty, and fragmented surveillance, the burden of disease attributable to harmful algal blooms exposure remains unclear. This scoping review aimed to map surveillance and data collection mechanisms, and inter-organisational data flows concerning marine harmful algal bloom-related health outcomes in the Philippines, Malaysia and Indonesia. The review followed Arksey and O’Malley’s framework and the Preferred Reporting Items for Systematic reviews and Meta-Analyses extension for Scoping Reviews reporting guidelines, searching PubMed, Web of Science and Scopus (1970–2024) and complementary searches in Google Scholar. Data extraction focused on intoxication types, data sources, reporting mechanisms and stakeholder collaborations. Twenty-three studies and records were included: 70% from the Philippines, 26% from Malaysia and 4% from Indonesia. Five main surveillance data sources were identified: (i) physician reports, (ii) healthcare facility records, (iii) local health authorities, (iv) Department of Health systems and (v) document-based and media-based data sources. However, key data infrastructure was poorly documented. The Philippines demonstrated the most diversified data pathways, emphasising multi-stakeholder collaborations. This review reveals that the infrastructure for harmful algal bloom-related health surveillance across the countries is largely absent or undocumented. Strengthening data systems and enhancing multi-sectoral collaboration are essential to mitigate the health threats posed by harmful algal blooms.

## Highlights

1. Most records came from the Philippines; Malaysia and Indonesia were underrepresented.
2. Identified data sources included physician reports, facility records and government data.
3. Malaysia relied on physician networks; others showed more diverse, multi-actor flows.
4. Key public health system features (e.g., disease coding and data integration) were not documented in any included record.
5. Weak surveillance systems hinder disease burden estimation and timely harmful algal bloom-related health responses.

## Introduction

The interdependence between marine ecosystem health and human health is examined within the interdisciplinary field of Oceans and Human Health (OHH), which is providing growing evidence that ocean and human health are inextricably linked, and that human interactions with marine environments will significantly shape future planetary outcomes [1].

Within this framework, harmful algal blooms (HABs) represent a key pathway linking ocean processes to human health risks. HABs are proliferation events in which certain species of microscopic algae – including dinoflagellates, diatoms and cyanobacteria – rapidly multiply in marine, brackish or freshwater environments. They are increasingly recognised as a major ecological concern [2–5] and as a defining health hazard alongside chemical pollution, microbial contamination and climate-driven ecological change [6,7].

HABs can affect human and animal populations, through direct toxin production or by altering ecological conditions such as oxygen depletion that impact marine resources [8]. Indeed, HABs degrade water quality and promote toxin accumulation in fish and shellfish, posing significant food safety risks and causing a range of human intoxications. These include amnesic (ASP), azaspiracid (AZP), ciguatera (CP), diarrhetic (DSP), neurotoxic (NSP) and paralytic shellfish poisoning (PSP), as well as palytoxicosis and *Ostreopsis* spp.-related syndromes (see Table 1 for a comprehensive description of clinical manifestations per syndrome, and refer to the original table therein).

**Table 1.** List of included seafood intoxications, with relative exposure routes to humans and toxins, readapted from Berdalet et al. (for a comprehensive description of clinical manifestations per syndrome, refer to the original table therein) [7]

Intoxication	Exposure route to humans	Major toxin	Symptoms	Reported blooms in Southeast Asia	Clinical diagnosis method
Amnesic shellfish poisoning	Shellfish	Domoic acid (DA)	Gastrointestinal, neurological	Yes [7,9–11]	Presumptive clinical diagnosis; no point-of-care test. DA detectable in human urine by liquid chromatography-tandem mass spectrometry (LC-MS/MS) (epidemiological use) [12].
Azaspiracid shellfish poisoning	Shellfish	Azaspiracid toxins	Gastrointestinal, neurological	No evidence found	Presumptive clinical diagnosis; no point-of-care test. No validated human biomarker; diagnosis relies entirely on clinical presentation [13].
Ciguatera poisoning	Coral reef fish and invertebrates	Ciguatoxins	Gastrointestinal, neurological, cardiovascular (can persist for several months or even years)	Yes [14,15]	Presumptive clinical diagnosis; no point-of-care test. No validated human biomarker at any level [13,16].
Diarrhetic shellfish poisoning	Shellfish	Okadaic acid	Gastrointestinal, neurological, respiratory	No evidence found	Presumptive clinical diagnosis; no point-of-care test. No validated human biomarker; diagnosis relies entirely on clinical presentation [17].
Neurotoxic shellfish poisoning	Shellfish and inhalation of blooms	Brevetoxins	Gastrointestinal, neurological, inflammatory	Yes [18,19]	Presumptive clinical diagnosis; no point-of-care test. Brevetoxin metabolites detectable in human urine/serum by enzyme-linked immunosorbent assay or LC-MS/MS (research-grade, not routine) [20,21].
Palytoxicosis and *Ostreopsis* spp.-algal syndrome	Fish and marine aerosols	Palytoxins	Gastrointestinal, neurological, cutaneous	Yes [22,23]	Presumptive clinical/epidemiological diagnosis; no point-of-care test. No validated human biomarker; diagnosis supported by environmental cell counts and bloom exposure history [4].
Paralytic shellfish poisoning	Shellfish, crustacean, fish	Saxitoxins	Gastrointestinal, neurological, mortality	Yes [18,24]	Presumptive clinical diagnosis; no point-of-care test. Saxitoxin detectable in human urine by LC-MS (epidemiological use) [25,26]

Clinical manifestations vary, most commonly involving: gastrointestinal (e.g., nausea, vomiting, diarrhoea, abdominal cramps, fever, chills, increased salivation); neurological (e.g., paraesthesias, memory loss, seizures, temperature reversal dysaesthesia, confusion, dizziness, ataxia, headache, drowsiness, dysphagia, blurred vision, tremor); respiratory (e.g., asthma-like symptoms, bronchoconstriction, respiratory paralysis, mucosal irritation, nonproductive cough, shortness of breath) and cardiac effects (e.g., labile blood pressure, bradycardia, hypotension, arrhythmia). In some cases, symptoms may persist for months or years and can be fatal. Table 1 also shows that across all seven syndromes, diagnosis relies on presumptive clinical assessment based on symptom presentation and exposure history, with no validated point-of-care test available for any of them. Analytical detection of the causative toxins in humans exists only for ASP, NSP and PSP, and remains limited to epidemiological investigations rather than routine clinical use. Furthermore, although some blooms are visible as surface water discolouration (‘red tides’), many occur without observable changes, requiring laboratory-based detection of environmental samples.

Assessing the burden of disease (BoD) of HAB-related illnesses remains challenging. It calls for robust evidence, based primarily on disability-focused, rather than mortality-based, data [6,7,14,16,27,28]. The estimation is affected by limited awareness, unclear diagnoses and scarce integration of surveillance and monitoring within health systems [2,6,16], with cases often misdiagnosed by and/or unreported to health authorities, a gap that joint Food and Agriculture Organisation/World Health Organisation (FAO/WHO) consultations on ciguatera alone have estimated leaves 80–90% of cases formally unrecorded, prompting explicit calls for strengthened epidemiological surveillance [29].

Marine HAB-related intoxications have been documented in Southeast Asia since the 1970s, particularly in the Philippines, Malaysia and Indonesia, with the available evidence recognising the first two as the most severely affected countries in the region [14,30,31]. In these settings, millions of individuals rely on the marine ecosystem for their livelihood and sustenance [5,32,33] making them especially vulnerable to the health, social and economic impacts of marine environmental change [34]. The capacity to generate reliable data on marine HAB-related illness depends on national health system structures, which shape governance arrangements, service delivery and reporting pathways. The Philippines operates a devolved system in which local government units are responsible for reporting and care, with geographical and workforce constraints limiting coverage across its archipelago [35,36]. Malaysia has a dual public–private system, recently reformed through the 2023 Health White Paper, where community clinics (klinik kesihatan) serve as the main primary care entry point [36,37]. Indonesia’s decentralised system is anchored in the Jaminan Kesehatan Nasional (JKN), achieving near-universal coverage since 2014, but with persistent disparities across remote coastal and island areas [37,38]. Across all three countries, community health centres are the first point of contact for marine HAB-exposed populations, yet differ in diagnostic capacity, laboratory access and reporting practices.

Accordingly, HAB-disease surveillance reflects how these systems are organised and coordinated. All three countries maintain national surveillance structures (namely, the Philippine Integrated Disease Surveillance and Response – PIDSR; Malaysia’s Communicable Diseases Control Information System – CDCIS; Indonesia’s Early Warning Alert and Response System – EWARS/SKDR), known to be scattered across many health outcomes, each facing challenges in capturing HAB-related illness. In the Philippines, PSP is notifiable within the PIDSR and the National Epidemic Sentinel Surveillance System (NESSS), yet other HAB-associated illnesses endemic to the region, such as ciguatera, fall outside these reporting frameworks [39]. Meanwhile linkages with environmental monitoring by the Bureau of Fisheries and Aquatic Resources (BFAR) remain limited [40,41]. In Malaysia, monitoring is long-standing but largely concentrated in the state of Sabah [42], and intoxications are not nationally notifiable as a distinct category. None of the non-communicable disease surveillance systems in Malaysia identified by Chandran et al. in their 2021 review integrate systematic reporting of HAB-related illness at the national level [43]. Currently Indonesia also has no dedicated HAB-disease surveillance mechanism [44]. The country’s decentralised health system comprises over 40 national-level surveillance programmes and more than 10,000 reporting facilities, but data collection practices remain heterogeneous and community participation uneven [45]. Recent efforts to strengthen the EWARS through a WHO–Ministry of Health collaboration (2023) may offer a future entry point for HAB-related illness reporting [46].

These limitations are further influenced by the absence of specific International Classification of Diseases (ICD)-10 codes, with PSP variably classified under broad toxic effect categories (T61.1, T61.2, T61.8), constraining case identification in health records and mortality data [47,48], while ICD-11, although more detailed, is not yet consistently implemented [49]. These elements operate as a cascade rather than as parallel barriers: diagnostic uncertainty constrains the assignment of dedicated disease codes; the absence of dedicated codes constrains case aggregation across reporting nodes and sectors; fragmented reporting in turn constrains BoD [50]. Healthcare-seeking behaviour acts as a gatekeeper in the cascade upstream, determining which cases enter the clinical system at all [50].

The reliance of these countries’ health and well-being on marine ecosystems [51] makes it crucial to implement adequate management strategies for coastal areas. Identifying which systems are, or have been, active in tracking HAB-related diseases provides the empirical basis for improved health data management, supports research, informs risk assessments, reveals gaps in surveillance [52] and strengthens public health responses [53].

Therefore, the aim of this scoping review was to identify existing data collection systems, nodes and data flows on human health conditions that result from the exposure to marine HABs in the Philippines, Malaysia and Indonesia. Where possible, we reconstructed the organisational collaborations underpinning data collection flows among various actors within the health systems of these countries, including communities, healthcare providers, local authorities and national authorities.

## Methods

Our methodology followed the 5-stage framework by Arksey and O’Malley and the Preferred Reporting Items for Systematic reviews and Meta-Analyses extension for Scoping Reviews (PRISMA-ScR) Checklist [54], and built on Young et al.’s 2020 Systematic Scoping Review on HABs and human health [27], as described below. The study protocol was registered at Open Science Framework [55]. This protocol is aligned with the broader effort in environmental health research to employ transparent systematic review methodologies where appropriate [56].

### Literature search

The search explored PubMed, Web of Science (WoS) and Scopus from 1970 [14] to 23 July 2024 (search date) to retrieve studies reporting methods used to investigate: marine HAB-related human intoxications (amnesic shellfish poisoning; azaspiracid shellfish poisoning; ciguatera poisoning); diarrhetic shellfish poisoning; neurotoxic shellfish poisoning; PSP, palytoxicosis and *Ostreopsis* spp.-algal syndrome, in the Philippines, in Malaysia and in Indonesia.

The search string (Appendix A) was built around synonyms for: geographical locations; human populations of the Philippines, Malaysia and Indonesia; the seven human intoxications, their toxins and vectors.

#### Complementary searches

Complementary searches were conducted to identify grey literature, policy documents, institutional reports and other records not captured in bibliographic databases, following the methodology adopted in Chandran et al.’s 2021 review [43].

Specifically for Indonesia, due to the absence of results from the systematic search, one author (R.P.) conducted a targeted Google Scholar search in November 2024. This search aimed to identify studies reported in Bahasa Indonesia using the keywords ‘*keracunan* [Indonesian for poisoning] *harmful algal blooms* Indonesia’ and reviewing the first 10 pages of the search results.

For the Philippines and Malaysia, broader searches were performed in Google Scholar, covering the period from 1970 to 23 July 2024. Search strings were intentionally broad to capture limited or poorly indexed documentation, including records in local languages. Intoxication syndromes were grouped into four clusters based on expected regional documentation: PSP, ciguatera, other shellfish poisoning syndromes and rarer HAB-related intoxications. A local-language string was added per country to capture grey literature not indexed in English. The full strategy is reported in Appendix A. For feasibility, screening was limited to the first 10 pages of results for each Google Scholar string.

Additional hand searches were conducted to identify publicly available online documents, with results screened according to the relevance ranking delivered by search engines. Targeted searches of relevant organisational websites were also performed to retrieve grey literature, including reports, guidelines, policy documents, advisories, surveillance materials and programme documents.

#### Complementary dataset consultation

The authors also browsed targeted websites of relevant organisations for grey literature. Websites of these organisations were searched for potentially relevant documents (reports, guidelines, policy and programme documents). To verify whether HAB-disaggregated case-level data existed beyond the records retrieved by the systematic search, all main national and supranational datasets potentially holding such information were directly consulted. The datasets queried were the Philippines’ Field Health Service Information System (FHSIS), the Philippine Integrated Disease Surveillance and Response (PIDSR) Weekly Surveillance Reports and the Department of Health Freedom of Information (FOI) portal; the Intergovernmental Oceanographic Commission-United Nations Educational, Scientific and Cultural Organization (IOC-UNESCO) Harmful Algal Event Database (HAEDAT); Malaysia’s CDCIS and the Ministry of Health’s Health Facts publications; Indonesia’s Badan Pengawas Obat dan Makanan (BPOM) Sentra Informasi Keracunan Nasional (SIKERNAS), the Early Warning Alert and Response System (EWARS/SKDR) and the National Health Insurance Sample Data (NHISD) held by BPJS Kesehatan; the ASEAN Rapid Alert System for Food and Feed (ASEAN RASFF) and the joint FAO/WHO International Food Safety Authorities Network (INFOSAN). This consultation was descriptive in scope and did not contribute records to the data extraction stage; its purpose was to document the accessibility of HAB-related case data across these sources.

### Inclusion and exclusion criteria

Inclusion criteria replicated those applied in Young et al.’s Systematic Scoping Review (2020) [27], with the focus on the Southeast Asian countries of the Philippines, Malaysia and Indonesia. Within these criteria, the review’s operational scope was framed as the surveillance of ingested ichthyosarcotoxins relevant to marine HAB-related syndromes. These toxins are acquired through seafood consumption that share clinical presentation pathways, healthcare-seeking patterns and surveillance architectures with HAB-derived intoxications.

Given the comprehensiveness of our aim, we adopted an inclusive approach to study designs and report types. Thus, our analysis included peer-reviewed studies, conference proceedings, non-peer-reviewed reports, discussion papers, abstracts, commentaries, media reports, etc. following the criteria in Table 2.

**Table 2.** Inclusion and exclusion criteria applied for record screening

	Inclusion criteria	Exclusion criteria
Population	Human population reported in the Philippines, Malaysia and Indonesia	Non-human; human cell lines; in vitro Studies not in the Philippines, Malaysia and Indonesia Studies on Filipinos, Indonesians and Malaysians treated elsewhere
Exposure	Microalgae or micro-algal derived toxins (bloom event not required) Routes: ingestion; inhalation; skin contact; sexual transmission; eye contact; other	Exposure via desalinated water; experimental exposure; food supplements Exposure to palytoxin via coral/cnidarians/zoanthids Seafood-consumption intoxications from other chemical and biological contaminants
Context	Marine, estuarine, coastal	Freshwater including lakes and rivers (if not specifically related to estuaries)
Outcome	HAB-related human intoxications listed above Other adverse health effects including but not limited to: death, gastrointestinal illness, respiratory illness, neurological illness, hospital admissions	Economic and societal impacts Presence of HAB events and toxins, without reporting human intoxications
Study characteristics	English language 1970–23 July 2024 (search date) Data source(s) used for the study are reported Real world data included as input variables for modelling purposes	Non-English language Publication prior to 1970 Reviews (set aside for backward citation screening) Modelling studies using simulation data as input variables Studies reporting HABs intoxication cases without data source information

From the final pool of articles, backward citation screening was conducted to make sure that no relevant source had been lost.

### Study selection

All references retrieved by the initial searches were collected and de-duplication was performed. Four authors (A.M., N.S., G.L., R.P.) screened against inclusion and exclusion criteria in a multi-stage manner. Disagreements were solved through discussion or by involving a fifth investigator.

Titles and abstracts were independently screened, followed by a full-text review via library access. Exclusion reasons were recorded during in-depth screening using Rayyan (Rayyan – AI Powered Tool for Systematic Literature Reviews). The study selection process has been documented in a PRISMA flow diagram (Fig. 1).

**Figure 1:**
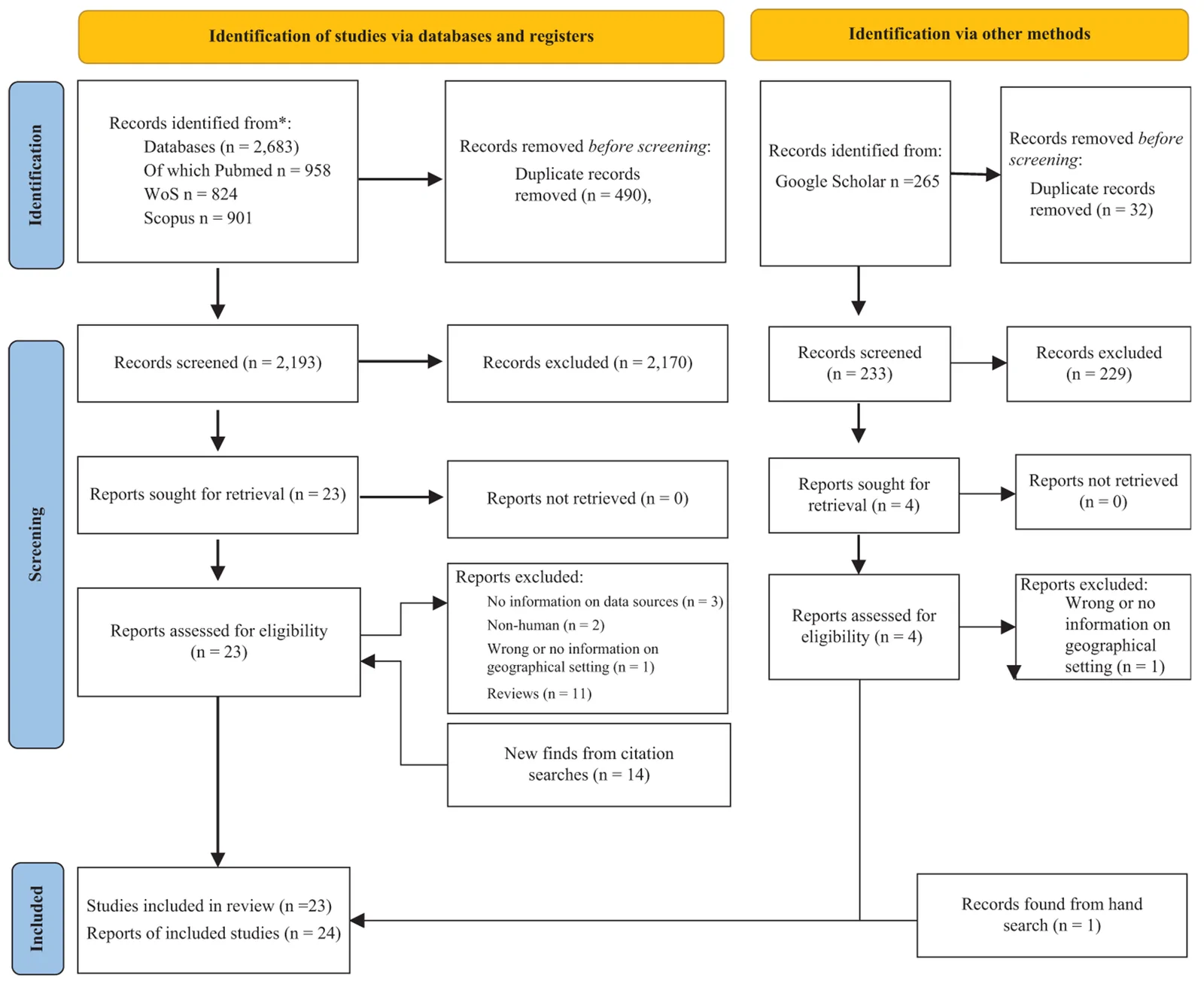
PRISMA flow chart of included studies.

### Data extraction

Data were extracted independently by four investigators (A.M., N.S., G.L., R.P.) using a standardised Excel form. Information was grouped into three domains: record identification, intoxication characteristics and data source characteristics (Appendix B).

### Data synthesis

Descriptive statistics were presented (counts and percentages, mean/median with standard deviation/interquartile range, as appropriate) for all the quantitative items using STATA software for Windows, v.16.1 (Stata Corp., College Station, TX, USA, 2019). Other relevant results (e.g., points of data collection, data flows, stakeholders involved and relevant relationships among them) were presented narratively, differentiated for each country and supported by visual tools (i.e., maps, tables and flow charts).

## Results

The search strings led to 2683 records from the PubMed, Scopus and WoS databases. After de-duplication, 2193 records were screened by title, abstract and full text. Backward citation screening was conducted on the reviews, and 20 records met the eligibility criteria and were included in this review for data extraction (see Supplementary Table – data extraction). Three articles were retrieved by means of the targeted complementary searches performed on Google Scholar (Fig. 1).

Moreover, the complementary grey literature searches yielded two additional papers while the hand searches retrieved one institutional document by the same author [57] which complemented some additional information on the already included record by Ching et al. [58].

Interestingly, none of the national or supranational datasets consulted yielded extractable, HAB-disaggregated case-level data on the seven syndromes investigated. The access constraints identified are summarised in Appendix C.

### Data extraction

Among the 23 reports included [18,42,58–78] (Supplementary Table), published from 1977 to 2018, six (26%) [42,70,72–75] were set in Malaysia; 16 (70%) [18,58–69,71,77,78] were set in the Philippines and one (4%) was set in Indonesia [76] (Fig. 2).

**Figure 2:**
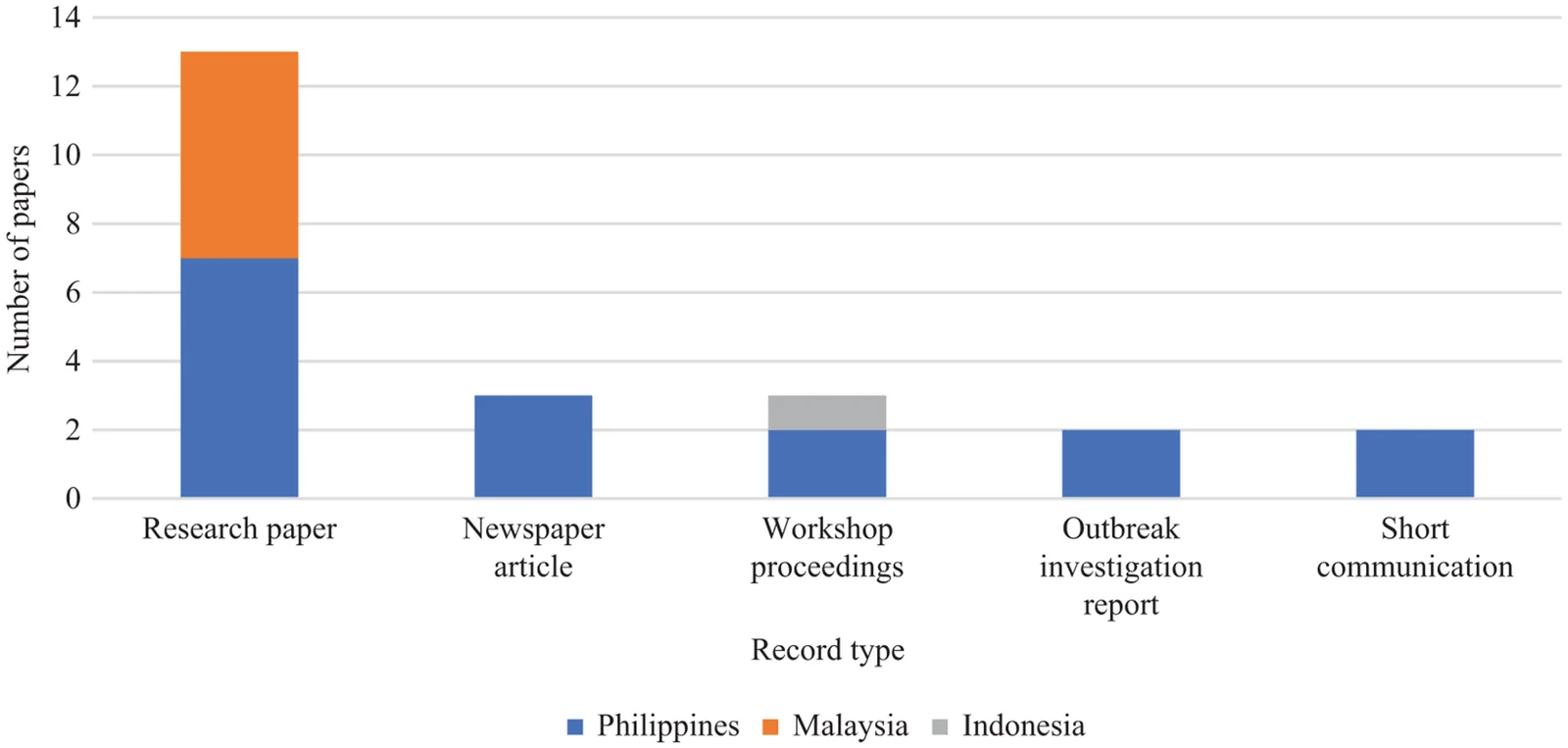
Distribution of records per type and country.

In terms of record type, 13 (56%) [18,42,59–61,65,70–75,77] were research papers, three were newspaper articles (13%) [62,66,68], three were workshop proceedings (13%) [64,67,76], two were outbreak investigation reports (9%) [58,69] and two were short communications (9%) [63,78] (Fig. 2).

Concerning the study design, case series were the most common, with 15 studies [18,42,59,61,63,64,67,69–75] accounting for 75% of the 20 studies (not including news reports). The remaining include: one laboratory-based experimental study [60], one employing mixed-methods [58] (combining descriptive methods for case identification and review, case-control analysis, laboratory testing and environmental assessment), one case-control [76], one mixed-method historiographic synthesis [77] and one review. Despite being a review, the 1994 paper by Hartigan-Go and Bateman [65] was included in the final synthesis for two reasons: firstly, three out of four of its original papers were not retrievable elsewhere; moreover, some information concerning the data sources of the outbreaks was not present in the original papers.

Of the seafood intoxications reported, 10 papers (43%) addressed PSP linked to crabs or bivalves [42,58,60,61,64,65,67,71,76,77]; another six (26%) focused on ciguatera from barracuda, red snapper or mackerel [18,61,68,69,71,73]; and four (17%) [72,74,75,77] described tetrodotoxin (TTX) from diverse organisms. Another three (13%) reported generic crab-related intoxications [59,63,70]. Two [62,66] (9%, both news reports) did not specify the toxin-type but involved barracuda or green mussels. One reported ‘red tide poisoning’ generically [78]. The average number of cases per study was 102.6, with half reporting fewer than 22 cases.

No records were found for amnesic, azaspiracid, diarrhetic and neurotoxic shellfish poisoning, nor for palytoxin poisoning and *Ostreopsis* spp. algal syndrome.

Five main categories of data sources were found: (i) physician-provided reports and personal communications from families, (ii) healthcare facility records, (iii) local health authorities, (iv) Department of Health (DoH) data, and (v) document- and media-based data sources. These categories structure the Results section, presented separately for Malaysia, the Philippines and Indonesia, and summarised in Table 3, which also notes instances of multi-stakeholder data collection.

**Table 3.** Categorisation of data sources

First author, year	Country	Intoxication	Data source category	Collaborative data flow (if reported)
Alcala et al. (1988) [59]	Philippines	Crab poisoning	Healthcare facility records	Not reported
Asakawa et al. (2010) [60]	Philippines	PSP	Physician-provided report and personal communication from families of victims	Not reported
Azanza et al. (2019) [71]	Philippines	PSP and ciguatera	Document-based and media-based data sources	Not reported
Azanza (2006) [61]	Philippines	PSP and ciguatera	DoH data	Not reported
Ching et al. (2015) [58]	Philippines	PSP	Healthcare facilities, DoH data	Healthcare facilities, national institutions and the community
De Leon-Bolinao and Ambrosio (2016) [77]	Philippines	PSP and TTX	Document-based and media-based data sources	Healthcare facilities and national institutions
Gabieta (2013) [62]	Philippines	Not specified	Non-clinical administration report	Not present
Gonzales and Alcala (1977) [63]	Philippines	Crab poisoning	Physician-provided report and personal communication from families of victims	Not present
Gonzales (1989) [64]	Philippines	PSP	Physician-provided report, local health authorities, healthcare facilities, DoH data	Healthcare facilities, local authorities and national institutions
Hartigan-Go and Bateman (1994) [65]	Philippines	PSP	Healthcare facilities, local health authorities, DoH data	Healthcare facilities, local authorities and national institutions
Kan et al. (1987) [72]	Malaysia	Pufferfish poisoning	Physician-provided report	Not reported
Lee et al. (2019) [73]	Malaysia	Ciguatera	Physician-provided report	Not reported
Loke and Tan (1997) [74]	Malaysia	Pufferfish poisoning	Physician-provided report	Not reported
Lyn (1985) [75]	Malaysia	Pufferfish poisoning	Physician-provided report	Not reported
Mendoza et al. (2013) [18]	Philippines	Ciguatera	Healthcare facility records, document-based and media-based data sources	Not reported
Nurlina et al. (2018) [76]	Indonesia	PSP	Regional Health Office, community health clinics, Ministry of Marine and Fisheries Affairs	Local health authorities, research institutions, national authorities and the community
Pareño (2014) [66]	Philippines	Not specified	Physician-provided report	Not reported
Pastor et al. (1989) [67]	Philippines	PSP	DoH data	Not reported
Ponsaran-Rendon (2010) [68]	Philippines	Ciguatera	DoH data	Not reported
Rebato et al. (2018) [69]	Philippines	Ciguatera	Healthcare facilities, DoH data	Healthcare facilities and national institutions
Sotto (1995) [78]	Philippines	‘Red tide poisoning’	Healthcare facilities, DoH data	Healthcare facilities and national institutions
Suleiman et al. (2017) [42]	Malaysia	PSP	Physician-provided report	Not reported
Suleiman et al. (2017) [70]	Malaysia	TTX (crab-related)	Physician-provided report	Not reported

*with integration from Ching [57].

Notably, no record entirely described the data sources used, thus it was often not possible to retrieve the following information: type of case finding (i.e., active vs. passive), financing sources, frequency of collection, key actors in charge of alerts and collection, collection systems, storage infrastructure, data recipient, data accessibility, disease coding methods, whether the consulted database was part of a broader monitoring network, of a temporary programme/intervention or whether the data were used for periodical elaboration of indicators. When retrievable, this information was inserted in the data source/case documenting column of the data extraction table.

#### Malaysia

In Malaysia, all cases [42,70,72–75] were reported by healthcare providers directly, therefore falling under the category of ‘physician-provided reports’. There was no indication of whether case recording on behalf of the healthcare facilities followed routine, systematised recording and reporting practices. One case series [72] on TTX poisoning in 18 people also involved a field investigation at victims’ homes, although no further details were reported.

#### Philippines

The 16 records from the Philippines [18,58–69,71,77,78] showed a wider variety of data sources compared to Malaysia. They included a mix of direct personal communications from families to authors, physician-provided reports, administrative reports, clinical and hospital records and government investigations.

##### Personal communications and physician-provided reports

Five records (31%) [60,62–64,66] relied wholly or partly on direct individual reports, mostly from family members or attending physicians directly to authors. In one case [62], the source was the Samar provincial agriculturist.

##### Healthcare facilities’ records

Two studies (9%) used hospital records. Specifically, one was a single case report on a crab poisoning [59], while the other described a ciguatera outbreak involving 22 cases [18].

##### DoH data

Three records [61,67,68] (19%) relied on data from the DoH. In 1989, the DoH found 437 PSP cases by means of questionnaires administered to suspected cases [67]. Second, Azanza [61] utilised data from the Field Epidemiology Training Program (FETP) supplemented by DoH health advisories, to understand 60 foodborne outbreaks between 1995 and 2004, including ciguatera and PSP. Lastly, a 2010 news report [68] was based on an investigation led by the DoH’s Centre for Health Development VI Regional Epidemiology Center in Iloilo, which uncovered 22 ciguatera cases.

##### Document-based and media-based data sources

One study [71], broadly examining food intoxications in the Philippines from 2005 to 2018, used data from multiple sources such as web portals, electronic archives of local news agencies and newspapers, government websites and reports from the Epidemiology Bureau of the DoH. However, the study did not specify which sources informed the seafood intoxication data specifically.

##### Collaborative data flows

Six studies (38%) [58,64,65,69,77,78] reported collaborative data collection involving communities, healthcare providers and local and national authorities.

##### Healthcare facilities and local authorities

Two records overviewed outbreaks in the Philippines from the early 1980s to the early 1990s, with one being a workshop proceeding [64] and the other a review [65]. Both records referred to outbreaks in Samar in 1983. Gonzales [64] reported a provincial hospital physician seeing 30 PSP cases per day, although the outbreak’s duration was unclear. Additional reports came from the Integrated Provincial Health Office and a district hospital, but total case numbers remain unknown. According to the 1994 review [65], a follow-up investigation was launched by the Provincial Health Office with the Ministry of Health’s regional office, although no record was identified.

##### Healthcare facilities and national institutions

Rebato et al. [69] described a collaboration between the DoH and the treating hospital. After the Epidemiology Bureau received an unspecified report of eight suspected ciguatera cases among seafarers, an event-based investigation was launched. The hospital’s surveillance officer identified cases from admission records; and interviews were conducted to collect demographic, clinical and food-related information, including preparation of the contaminated barracuda. In their 1995 short communication, Sotto [78] also described a parallel configuration centred on the Red Tide Task Force (RTTF), a joint Department of Agriculture-DoH body that reported PSP case counts, deaths and hospital admissions to the Health Secretary. Cases were updated through official and hospital reports, complemented by shellfish toxin test results exceeding allowable limits.

##### The community and national institutions

Gonzales [64] reported that in 1987, an officer from the BFAR mussel farm in Zambales, who was trained in red tide detection, identified two cases of PSP and alerted the agency’s Research Division. The Integrated Provincial Health Office and a district hospital reported nine and four additional cases, respectively.

##### Healthcare facilities, national institutions and the community

An outbreak investigation [58] was led in July 2013 after the National Epidemiology Centre’s Event-Based Surveillance & Response Unit received a report for PSP. A team from the DoH then identified 31 cases by reviewing medical records from the local health centre and district hospital. Active case finding was also conducted in the two affected villages, including interviews with relatives of deceased individuals.

##### Healthcare facilities, local authorities and national institutions

Among the series of outbreaks in 1988 and 1989 reported by Gonzales [64], 24 cases of PSP from one district hospital in Cebu sparked an epidemiological investigation by the DoH. Furthermore, 45 cases were recorded in Leyte by the DoH Regional Office. According to the 1994 review by Hartigan-Go and Bateman [65], the FETP was also sent to identify populations at risk, involved vectors, the mussels’ provenance and poisoning patterns.

In February 1991, 11 cases were admitted to the provincial hospital in Camiguin. The outbreak was discovered by BFAR and reported to the FETP 10 days after the patients’ admissions. In August, the FETP conducted ulterior on-site investigations in Bataan, Cavite and Manila, uncovering 66 additional cases that had occurred between July and August 1991.

##### Multisectoral inter-agency bodies, healthcare facilities and media-based documentation

De Leon-Bolinao and Ambrosio’s mixed-methods historiographic synthesis describes the RTTF issuing weekly Red Tide Updates, a Department of Agriculture–Department of Health joint serial publication reporting DoH-channelled case counts and fatalities [77]. The Inter-Agency Committee on Environmental Health (IACEH), released periodic press releases consolidating outbreak case counts and deaths from the DoH while coordinating with multisectoral partners across fisheries, science, environment, local government, communication and maritime sectors. These institutional sources were complemented by provincial and district hospital admission records and approximately 28 newspaper articles documenting case counts, fatalities and hospitalisations across the period. The record also draws on four interlinked sources documenting PSP outbreaks between 1988 and 1996, which partly overlap with those reported by Gonzales in 1989 [64], Pastor et al. in 1989 [67] and Hartigan-Go and Bateman in 1994 [65]. It was retained for its documentation of institutional and structural data sources not captured elsewhere in this review.

#### Indonesia

##### Collaborative data flow involving local health authorities, research institutions, national authorities and community

In Indonesia, a post-event investigation [76] was launched after the Cirebon Regency Health Office received a written report from a community health centre surveillance officer on 33 food intoxication cases over two days. The authority alerted all surveillance officers via WhatsApp and Messenger (Meta Platforms Inc.) to report similar cases and initiate early warnings related to green mussel contamination. Researchers conducted follow-up interviews in affected villages, linking cases to laboratory-confirmed *Pyrodinium bahamense* blooms and saxitoxin levels reported by the Ministry of Marine and Fisheries Affairs’ Fish Disease and Environment Inspection Laboratory, ultimately identifying 115 cases, including two fatalities.

### The ecosystem of health data for HAB-related diseases

This section synthesises the review’s findings by contextualising them within the clinical pathway of patients with marine HAB-related intoxications. The flowchart in Fig. 3 reconstructs this ecosystem of health data and illustrates the actors involved in tracking the data flow, represented within the white boxes. The direction of the solid-line arrows represents the bottom-up case-reporting process, symbolised by an icon of a file. Conversely, the dashed-line arrows indicate possible top-down case-finding pathways, represented by a magnifying glass. Country flags indicate the identified actors involved in data flow across the studied countries.

**Figure 3:**
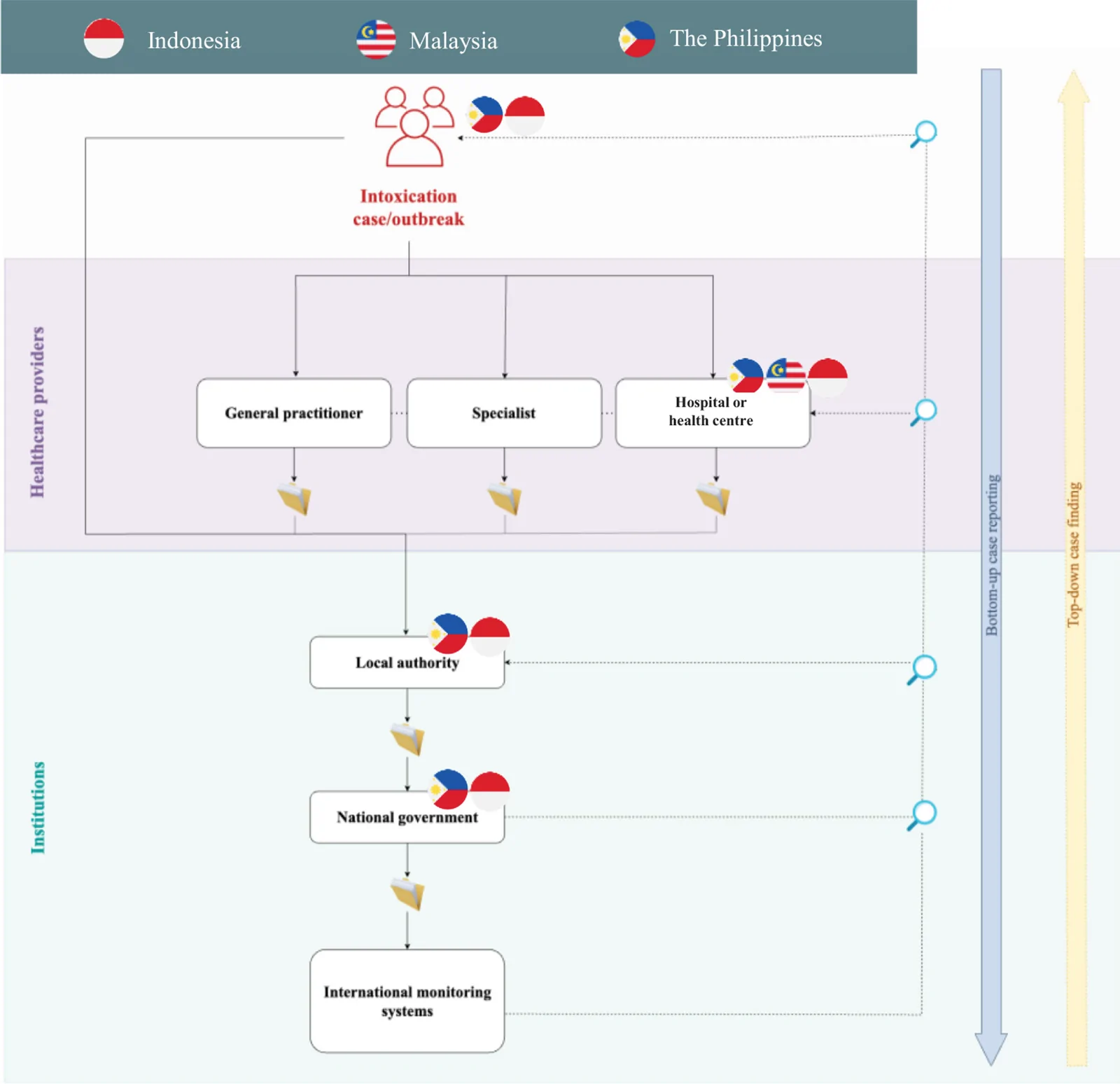
Ecosystems of health data: a visual exploration.

The data stream ‘nodes’ (i.e., where the occurrence of HAB-related diseases can be intercepted and recorded within the healthcare ecosystem) are not confined to a single point. Given the variety of healthcare settings necessary to treat HAB-related intoxications, these nodes span across the entire healthcare ecosystem, capturing data at multiple levels [16,20,79], including home care self-reports, primary care physician offices, local health authority records, outpatient ambulatory and inpatient acute care registries, pharmacies for chronic medication, and local and traditional carers.

Specifically, alerts originate directly from affected households or communities and are directed to local health authorities, provided that an appropriate communication channel exists and is known. Alternatively, cases enter the reporting system when individuals with symptoms seek medical care at general practices, specialist clinics or hospitals, where they are formally registered. The data may then be gradually centralised if passed on to institutions of increasing jurisdictional scope, such as regional authorities, central governments and, potentially, international monitoring systems, although no records were reported for the latter.

As shown, primary care (general practitioners) and outpatient secondary care (specialists) nodes were rarely identified as data sources in the included records.

## Discussion

The aim of this scoping review was to identify existing data collection systems, nodes and collaborative data flows in the Philippines, Malaysia and Indonesia on human health conditions that result from the exposure to marine HAB toxins.

### Investigated HAB-related diseases

Findings highlight a critical gap: there were no records of amnesic or neurotoxic shellfish poisoning, amnesic and neurotoxic shellfish poisoning, nor for palytoxin poisoning and *Ostreopsis* spp. algal syndrome, despite documented regional occurrence of the causative toxins (Table 1). In line with previous reviews investigating ocean-related human health concerns [80], most included records focused instead on ciguatera [18,61,68,69,71,73] followed by PSP [58,60,61,64,65,67,70,71,76,77] and TTX or organism-specific intoxications [59,62,63,66,70,72,74,75].

Although not HAB-derived, TTX was retained for several reasons. The review’s operational scope is the surveillance of ingested ichthyosarcotoxins relevant to marine HAB-related syndromes – toxins acquired through seafood consumption that share clinical presentation pathways, healthcare-seeking patterns and surveillance architectures with HAB-derived intoxications. Firstly, it is reasonable to assume they share monitoring systems with the other HAB-related intoxications. Secondly, excluding intoxications based on the HAB organisms could omit relevant data and geographical hotspots. Pufferfish TTX intoxication falls within this scope as a seafood-borne ingestion exposure that overlaps operationally with PSP, even though TTX and saxitoxin bind at distinct sites of the voltage-gated sodium channel and can in principle be differentiated by exposure history and toxin testing where available [60,81]. Realistic exposure scenarios also consider the presence of multiple toxins in a single organism, but current marine biotoxin regulations remain focused on single-toxin thresholds [82,83]. This gap may limit the accuracy of health risk assessments, as toxin interactions (additive, synergistic or antagonistic) can significantly alter toxicity [84]. Adopting mixture-based approaches could thus strengthen existing food safety regulations for HAB toxins. Lastly, evidence increasingly points to non-shellfish vectors as important saxitoxin sources [79,85] and vice versa for other intoxications. For example, recent recommendations require renaming ‘ciguatera fish poisoning’ to ‘ciguatera poisoning’ [20].

### Data sources and systems

Regarding data systems, types and sources, results showed substantial variability, highlighting gaps in regional surveillance and likely underreporting of marine HAB-related illnesses.

Firstly, the variability in data sources across the three countries echoes fragmentation. Malaysian data originate from physician-reported communications among healthcare providers, researchers and government officials. This reliance on generic channels for data collection raises concerns about the reliability, consistency and comprehensiveness of such data. In contrast, the Philippines exhibits a broader range of data sources, including healthcare facility records, DoH investigations, and collaborative data flows involving national and local authorities and communities. This diversity of sources is the result of a more structured approach to data collection, yet gaps remain in the understanding of reporting mechanisms, access and utility of the information. Lastly, despite the limited records available from Indonesia, the one resource identified [76] highlights a successful example of coordination and collaboration among multiple stakeholders, including local health authorities, communities and researchers, and a strong reliance on instant communication channels such as WhatsApp.

Second, none of the included records reported disease coding practices, reflecting the broader diagnostic uncertainty characterising HAB-related intoxications. As summarised in Table 1, for all HAB intoxications, diagnosis relies exclusively on presumptive clinical assessment based on symptom presentation and exposure history. No validated point-of-care test is available for any of them, while analytical detection of causative toxins in humans – where available at all – remains confined to epidemiological investigations rather than routine clinical use. Without a confirmatory test, distinguishing from other causes of gastroenteritis or neurological illness is time and resource consuming.

Moreover, the abovementioned lack of dedicated ICD codes means that even when cases are recognised, they are registered under generic categories such as food poisoning or the toxic effect of seafood, and therefore cannot be coded consistently in health records. This structural invisibility within health information systems has cascading consequences for surveillance.

The diagnostic gap is the root condition from which the rest of the surveillance cascade fails. Without validated case definitions, dedicated disease codes cannot be assigned consistently; without consistent codes, cases cannot be aggregated across reporting nodes or sectors; without integrated case data, burden-of-disease estimation remains structurally impossible. The pattern documented in this review – informal physician communications, mixed institutional pathways, single multi-stakeholder investigations – represents the visible surface of this cascade rather than a series of independent reporting failures. Healthcare seeking behaviour – an upstream determinant distinct from formal reporting – compounds this further: studies for ciguatera, conducted primarily in the Pacific, the United States and the Caribbean, document that fewer than 60% of affected individuals seek medical attention as many manage symptoms through self-care or traditional remedies [86]. No published data exist on care-seeking patterns for any of the other six syndromes, including in Southeast Asia. Precisely because the clinical entry point is inherently imprecise, the surrounding surveillance infrastructure (i.e., reporting pathways, multi-sectoral coordination and environmental monitoring integration) must be all the more robust to compensate.

Out of all the data items initially intended to be extracted, very few were found. Data source characteristics and collection methodology were seldom described, with no information retrieved on collection frequency, financing sources or the key actors responsible for issuing alerts and collecting data. Details on data accessibility – whether open or restricted – and storage infrastructure (paper-based, on-premise digital, cloud-based or centralised government servers) were similarly absent.

### Comparisons with existing monitoring systems and the importance of multi-sectoral collaborations

#### The structure of existing national health informative systems

In the investigated region, nation-wide health informative systems are mostly available (the PIDSR in the Philippines, CDCIS in Malaysia and EWARS/SKDR in Indonesia [44]) yet coverage in all three is constrained by service-delivery challenges in remote coastal areas, where HAB exposure is concentrated [44]. The challenge of delivery of healthcare services to remote areas could hinder a fair and equitable recording of health data [87,88].

Beyond these general systems, dedicated HAB programmes operate in all three countries but share a common architectural feature: surveillance is organised primarily around environmental hazards, with human health entering mainly as a response domain once an outbreak is declared rather than as a parallel routine surveillance stream.

In the Philippines, the Region VII RTTF, established after the 1992 *P. bahamense* bloom in Samar and Leyte, was framed as a decentralised alternative to a centralised national committee, mobilising the DoH, BFAR, local government units, the Coast Guard, the Philippine Ports Authority, Department of Science and Technology, universities and information and social welfare agencies. Yet its routine activities centred on phytoplankton counts, PSP testing in shellfish, restaurant and market sampling, shipment monitoring and clearance certificates; hospitals were ‘monitored for cases of PSP’ during outbreaks, but no formal case-reporting pathway, standardised case definition or routine linkage between clinical data and bloom/toxin monitoring was described. The institutional architecture described by these historical records has subsequently contracted. The HAB monitoring function is now operated by BFAR’s Fisheries Resources Management Division as a single-agency programme, with the Shellfish Bulletin replacing the earlier joint DA–DoH Red Tide Updates and DoH involvement reduced to event-based clinical response through regional Epidemiological Surveillance and Response Units. The IACEH, the DoH-chaired parent body that originally constituted the National RTTF, currently maintains five sectoral task forces – none of them marine. The multi-stakeholder configuration documented in Gonzales [64], Sotto [78], Ching et al. [58] and De Leon-Bolinao and Ambrosio [77] thus represents a historical institutional form rather than a current one. In Malaysia, the Sabah programme reported by Jipanin et al. [89] follows the same logic: the Department of Fisheries Sabah has led year-round monitoring at 69 stations under 13 district offices since 1976, while the MoH Sabah joins only once PSP cases are reported, by collecting samples for the fisheries laboratory and supporting public education materials. The Indonesian setting is illustrated by the PICES-MAFF-funded Ciguatera Indonesia project at Gili Matra, West Lombok [90], which integrates planktonic and benthic dinoflagellate sampling, SPATT-based ciguatoxin tracking, fish-tissue mouse bioassays and a socio-economic survey, but is project-based and research-led rather than institutionally embedded; the team’s own community survey documented that local fishing populations had never received any information on ciguatera despite rating such information as highly important, further underscoring the absence of a routine clinical or risk-communication pathway alongside the environmental work.

The surveillance logic across the three countries therefore remains largely hazard-led: blooms, shellfish toxicity and contaminated products trigger action, while case detection, reporting, classification and data integration on the human-health side remain fragmented or poorly documented. This supports our broader finding that monitoring capacity in the countries examined is more developed on the bloom and seafood-safety side than on the clinical surveillance side.

In this regard, the 2015 study by Ching et al. [58] describes an architecture that, if institutionalised, can enhance both surveillance and response. The Philippines’ DoH was deployed to investigate an outbreak. They identified 31 intoxicated cases through a review of medical records from outpatient clinics and admitted patients at the local health centre and district hospital, as well as through active case finding and interviews in the affected areas. This health-sector engagement translated into concrete environmental and policy actions when the local government of Tarangan was advised to ban shellfish harvesting in Cambatutay Bay (subsequently added as a sentinel site for dinoflagellate and saxitoxin monitoring). Alongside this, a community-led movement focusing on waste disposal practices and environmental clean-up drives was launched. These combined measures proved effective, with no further HAB events or PSP cases reported since the outbreak ceased. This serves as an excellent example of how the healthcare sector can actively participate in and influence risk mitigation and exposure reduction measures against such threats.

#### International policy mandates

Against the localised documented gaps, international frameworks and comparable surveillance architectures provide both a normative benchmark and concrete design options. The IOC–FAO Intergovernmental Panel on Harmful Algal Blooms (IPHAB), through its constituent strategies, has repeatedly urged Member States to develop capacity to monitor HAB-causing organisms, toxins, contaminated seafood and the associated epidemiology, including via Early Warning Systems integrated with the Global Ocean Observing System [91–93]. The joint FAO/WHO Expert Meeting on Ciguatera Poisoning reached a converging conclusion, identifying improved epidemiological surveillance as a priority for risk management given that only 10 to 20% of cases are estimated to be formally reported [29]. The surveillance configurations identified in our review fall short of these mandates.

Concerning international monitoring systems, some efforts for pooling information on HAB-related illnesses have indeed been made, i.e., the UNESCO Harmful Algae Informative System shows PSP and ciguatera events in the Philippines (UNESCO HAIS, n.d. and its sub-database HAEDAT). However, monitoring and surveillance systems for such diseases still are in their initial phases, presenting a low degree of penetration and outreach of data collection efforts.

Outside the investigated region, the US CDC’s HABISS (2007–2011) and its successor OHHABS (established in 2016) exemplify an integrated surveillance architecture capturing suspected human and animal cases alongside environmental bloom data within a single database, classified via purpose-built case definitions with severity tiers (suspected/probable/confirmed) rather than dependent on ICD coding [94,95]. Although currently US-only in scope, this One Health design may offer a transferable architecture for the three countries examined here – and one that addresses both the diagnostic-coding gap and the environmental–health integration gap identified by our review.

#### Implementation considerations

Nowadays, the accuracy of data collection is strongly dependent on the level of digitalisation of the countries and of the administrative areas within them. Unfortunately, functioning data collection nodes are easier to find in urban areas than in remote coastal zones, despite these rural areas often being more dependent on seafood consumption and more exposed to contact with the HAB toxins.

The utility of the recent transitions of the three countries to electronic health records (EHRs) [96] may improve reporting, although its impact will depend on the specificity of disease coding adopted for HAB-related intoxications as well as diagnostic recognition and healthcare accessibility – all gaps discussed above.

Often, information on toxin exposure routes is gathered through targeted public health initiatives (e.g., prevention campaigns, formal alert systems, hotlines, third party-funded programmes), when the urgency of specific situations heightens the need for collecting such data. Therefore, the nodes are more likely to emerge from locations under the spotlight of more influential stakeholders or arise from moments characterised by greater awareness or perceived impact. Such bias may be mitigated by evidence-based interventions derived from the fact that bloom monitoring (devoid of considerations regarding the actual impact on human health) is at more mature stages, a complementarity discussed in more detail below [97]. A strategic reorganisation of existing resources across the entire ecosystem of healthcare services could be an effective approach. The following considerations provide some insights as to where such reorganisations might occur. They are not intended to be exhaustive, as formulating a comprehensive intervention framework extends beyond the scope of our review.

Firstly, at the prevention level, community health literacy in coastal areas (covering red tide awareness, high-risk species and safe harvesting practices combined with systematic and timely public advisories and harvesting bans informed by bloom monitoring) constitutes a relatively accessible upstream intervention. Translating environmental signals into protective action, requires the kind of multi-sectoral collaboration between fisheries, environmental and public health authorities with the coordination of higher governance levels of the health system (i.e., Ministries of Health, national health councils and inter-regional institutions).

At the clinical interface, education and training of health professionals for diffused symptom recognition and structured exposure history-taking would allow for more defined presumptive clinical diagnosis. Moreover, primary care providers and community pharmacists represent additional underutilised nodes for early case recognition, particularly for milder presentations that do not reach hospital settings. This pattern is visible in Fig. 3: documented data sources cluster along the central reporting axis from healthcare facility to local authority to DoH, while peripheral nodes – primary care, outpatient specialists, pharmacies and traditional carers – appear in the conceptual ecosystem but were rarely identified as actual data sources in the included records. Activating these silent nodes is the concrete intervention target of the recommendations that follow.

A routine integration of HAB-related intoxication surveillance into existing national frameworks (PIDSR, CDCIS and EWARS) by using case definitions that are independent of laboratory confirmation – as, for example, operationalised by OHHABS – ensures a pragmatic approach to improved case recording, given current diagnostic constraints. Individual case identification and outbreak detection should be viewed as complementary rather than alternative objectives. For intoxications that cause chronic conditions, such as ciguatera, structured follow-up pathways within existing health facilities could reduce self-management and track disease progression in its manifestations beyond the acute phase. Finally, advocacy directed at national ministries and international classification bodies for dedicated ICD codes for HAB-related syndromes would generate cascading structural benefits; regional organisations such as ASEAN and IOC-UNESCO are well positioned to coordinate this effort. The UN Ocean Decade Harmful Algae Bloom Solutions (HAB-S) Programme, endorsed under IPHAB, offers a concrete and currently active vehicle through which Southeast Asian Member States could align national surveillance strengthening with the broader international framework, particularly with respect to data resources and decision-making for HAB-related human health impacts [98].

### Study strengths

To the best of our knowledge, this is the first scoping review applying a public health management lens to HAB-related intoxications by mapping existing data collection systems, nodes and collaborative data flows on human health conditions that result from the exposure to marine HABs, focusing on the public health networks of the Philippines, Malaysia and Indonesia. Given the novelty of this perspective, a systematic assessment of what had been formally documented was considered the most appropriate methodological starting point.

Beyond the mapping itself, the review reconstructs, when possible, the institutional collaborations involved in the flow of such data, among various health system stakeholders, including communities, healthcare providers, local authorities and national authorities. This aspect, often overlooked in existing HAB literature, clarifies the added value of integrated health system networks, and provides valuable examples for future organisational network design. Furthermore, it underscores the critical gap in formal reporting procedures and the lack of integration of health and environmental monitoring, contributing to the scarcity of data in this field.

### Limitations

The findings of this scoping review must be interpreted considering some limitations. Firstly, the methodological choice of a systematic scoping search of published literature may have excluded relevant data held in administrative databases, government records and other restricted or grey literature sources, potentially underestimating the number of existing data systems. To minimise this effect, citation screening was given considerable space – accounting for over 60% of included records – and inclusion criteria were deliberately broad, welcoming newspaper articles, outbreak investigation reports and other non-traditional record types. A complementary grey literature search was additionally conducted, although this yielded only three additional resources.

Secondly, the English language inclusion criterion for the systematic search may have excluded relevant local or national documents for Malaysia and the Philippines, although this may have been partially compensated through the complementary grey literature searches in Indonesian, Malaysian and Filipino. Lastly, many records on HAB-disease cases did not detail the data source used and were therefore excluded from our analysis. Thus, additional data sources may actually exist but could not be found by means of a systematic desk search alone.

Additionally, the protocol applied two source-based exclusions that may have shaped which records surfaced: palytoxin acquired via direct contact with corals, cnidarians or zoanthids – a non-ingestion exposure route falling outside the ingested-ichthyosarcotoxin scope of this review – and freshwater HAB toxins outside estuarine contexts. These exclusions are consistent with the review’s focus on seafood-borne exposures relevant to public health in coastal Southeast Asian populations, but records pertaining to these excluded exposure pathways were not assessed.

### Implications for future research and policy

Future research should aim to explore the presence and distribution of data collection nodes within the three Southeast Asian health systems through alternative research methodologies. This could include engaging local key informants to gather in-depth insights and conducting site visits alongside public health professionals to better understand contextual factors and opportunities. Supplementary records could be retrieved through formal consultation of national administrative databases via the institutional channels mandated for their access, as catalogued in Appendix C – a methodological path that requires in-country partnership but would capture data layers structurally inaccessible to systematic desk-based searches. Subsequently, implementation research will be essential to evaluate the feasibility and effectiveness of the surveillance strengthening approaches outlined in this review in real-world settings across the three countries [99].

HAB-induced intoxications sit precisely at the unaddressed intersection of Sustainable Development Goal (SDG) 14 (Life Below Water) [100] and SDG 3 (Good Health and Well-being) [101], and warrant priority attention across all three countries. The review’s findings, indeed, map onto three specific SDG targets: 3.d (‘strengthening national capacity for early warning, risk reduction, and management of health risks’), to which the proposed integration into PIDSR, CDCIS and EWARS directly contributes; 3.9 (‘reducing illness and mortality from hazardous chemicals and environmental contamination’), which places marine biotoxins in their proper aetiological frame; and 13.1 (‘resilience to climate-related hazards’) [102], relevant to the projected expansion of HABs under ocean warming. Naming these targets reframes the infrastructure gaps documented here as obstacles to commitments the three countries have already made. Within this picture, the near absence of Indonesian data is particularly concerning given philanthropic and intergovernmental investment in SDG 14.

Cross-border data-sharing across Southeast Asian countries – coordinated through existing regional bodies such as ASEAN and IOC-UNESCO – would strengthen response capacity as climate change alters HAB frequency and severity, while supporting food security in a region the WHO classifies as carrying an intermediate burden of foodborne disease [11,103].

## Conclusion

This scoping review documents that the infrastructure for marine HAB-related disease surveillance in the Philippines, Malaysia and Indonesia fails as a cascade, not as a set of independent gaps. The absence of validated clinical case definitions prevents consistent diagnostic recognition; the absence of dedicated disease codes prevents consistent case classification; the absence of integrated cross-sectoral reporting pathways prevents case aggregation – and together these compounding absences make credible burden-of-disease estimation structurally impossible.

These gaps persist despite an established intergovernmental call for action articulated through the IOC-FAO IPHAB and its constituent strategies [92,93,104]; the principal challenge is therefore no longer the absence of a policy mandate, but its national operationalisation. Closing these gaps through dedicated case definitions, integration into PIDSR/CDCIS/EWARS using laboratory-independent definitions modelled on OHHABS, advocacy for HAB-specific ICD codes, and structured linkage with environmental monitoring – is the prerequisite for credible burden-of-disease estimation and for translating the increasingly undeniable ocean–human health connection into protective public health action in Southeast Asia.

## Data Availability

All data generated or analysed during this study are included in this published article (and its supplementary information files).

## Appendix

### Appendix A

#### Search strings for PubMed

(‘Philippines’[Title/Abstract] OR ‘Luzon’[Title/Abstract] OR ‘Visayas’[Title/Abstract] OR ‘Mindanao’[Title/Abstract] OR ‘Palawan’[Title/Abstract] OR ‘Zambales’[Title/Abstract] OR ‘Pangasinan’[Title/Abstract] OR ‘Cavite’[Title/Abstract] OR ‘Pampanga’[Title/Abstract] OR ‘Bataan’[Title/Abstract] OR ‘Cambatutay’[Title/Abstract] OR ‘Metro Manila’[Title/Abstract] OR ‘Panay’[Title/Abstract] OR ‘Negros’[Title/Abstract] OR ‘Cebu’[Title/Abstract] OR ‘Bohol’[Title/Abstract] OR ‘Leyte’[Title/Abstract] OR ‘Samar’[Title/Abstract] OR ‘Masbate’[Title/Abstract] OR ‘Balete Bay’[Title/Abstract] OR ‘Balite Bay’[Title/Abstract] OR ‘Basilan’[Title/Abstract] OR ‘Bislig Bay’[Title/Abstract] OR ‘Casiguran’[Title/Abstract] OR ‘Aurora’[Title/Abstract] OR ‘Dauis’[Title/Abstract] OR ‘Davao’[Title/Abstract] OR ‘Davao Oriental’[Title/Abstract] OR ‘Davao Occidental’[Title/Abstract] OR ‘Hinatuan Bay’[Title/Abstract] OR ‘Lianga Bay’[Title/Abstract] OR ‘Litalit Bay’[Title/Abstract] OR ‘San Benito’[Title/Abstract] OR ‘Magueda Bay’[Title/Abstract] OR ‘Maguindanao’[Title/Abstract] OR ‘Santa Maria’[Title/Abstract] OR ‘Surigao’[Title/Abstract] OR ‘Honda Bay’[Title/Abstract] OR ‘Taytay’[Title/Abstract] OR ‘Malampaya Sound’[Title/Abstract] OR ‘Puerto Princesa’[Title/Abstract] OR ‘Taguines Lagoon’[Title/Abstract] OR ‘Camiguin Island’[Title/Abstract] OR ‘Canaco Bay’[Title/Abstract] OR ‘Bacoor Bay’[Title/Abstract] OR ‘Dumanguillas Bay’[Title/Abstract] OR ‘Murcielagos Bay’[Title/Abstract] OR ‘Zamboanga del Norte’[Title/Abstract] OR ‘Pilar’[Title/Abstract] OR ‘Sorsogon’[Title/Abstract] OR ‘Sual’[Title/Abstract] OR ‘Sapian Bay’[Title/Abstract] OR ‘Ivisan’[Title/Abstract] OR ‘Indonesia’[Title/Abstract] OR ‘Jakarta Bay’[Title/Abstract] OR ‘Kao Bay’[Title/Abstract] OR ‘Ambon Bay’[Title/Abstract] OR ‘Lampung Bay’[Title/Abstract] OR ‘Maninjau’[Title/Abstract] OR ‘Biak-Irian Jaya’[Title/Abstract] OR ‘Malaysia’[Title/Abstract] OR ‘Peninsular Malaysia’[Title/Abstract] OR ‘West Malaysia’[Title/Abstract] OR ‘Bachok’[Title/Abstract] OR ‘Johor’[Title/Abstract] OR ‘Kelantan’[Title/Abstract] OR ‘Kuantan’[Title/Abstract] OR ‘Malacca’[Title/Abstract] OR ‘Melaka’[Title/Abstract] OR ‘Pahang’[Title/Abstract] OR ‘Pantai Melawi’[Title/Abstract] OR ‘Sebatu’[Title/Abstract] OR ‘Sri Sebang’[Title/Abstract] OR ‘Sungai Geting’[Title/Abstract] OR ‘Teluk Bahang’[Title/Abstract] OR ‘Tumpat’[Title/Abstract] OR ‘East Malaysia’[Title/Abstract] OR ‘Malaysia Borneo’[Title/Abstract] OR ‘Sabah’[Title/Abstract] OR ‘Andus’[Title/Abstract] OR ‘Papar’[Title/Abstract] OR ‘Balambayan Island’[Title/Abstract] OR ‘Beaufort’[Title/Abstract] OR ‘Binnsuluk’[Title/Abstract] OR ‘Bongawan seashore’[Title/Abstract] OR ‘Gayana Resort’[Title/Abstract] OR ‘Inanam’[Title/Abstract] OR ‘Kota Kinabalu’[Title/Abstract] OR ‘Karambunai’[Title/Abstract] OR ‘Kg Kebagu’[Title/Abstract] OR ‘Kota Belud’[Title/Abstract] OR ‘Kota Marudu’[Title/Abstract] OR ‘Kuala Mengalong’[Title/Abstract] OR ‘Kuala menggatal’[Title/Abstract] OR ‘Kuala Penyu’[Title/Abstract] OR ‘Kuching’[Title/Abstract] OR ‘Likas Bay’[Title/Abstract] OR ‘Membakut’[Title/Abstract] OR ‘Miri’[Title/Abstract] OR ‘Numbak’[Title/Abstract] OR ‘Pelabuhan’[Title/Abstract] OR ‘Penampang’[Title/Abstract] OR ‘Penimbawan’[Title/Abstract] OR ‘Petagas’[Title/Abstract] OR ‘Pulau Gaya’[Title/Abstract] OR ‘Pulau Manukan’[Title/Abstract] OR ‘Pulau Sepangar’[Title/Abstract] OR ‘Rancangan Mawao’[Title/Abstract] OR ‘Sarawak’[Title/Abstract] OR ‘Sepanggar’[Title/Abstract] OR ‘Sepangar’[Title/Abstract] OR ‘Sg Mengkabong’[Title/Abstract] OR ‘Tanjung Aru’[Title/Abstract] OR ‘Tanjung Badak’[Title/Abstract] OR ‘Trayong’[Title/Abstract] OR ‘Tuaran’[Title/Abstract] OR ‘Serusup’[Title/Abstract] OR ‘Sipitang’[Title/Abstract] OR ‘Taman Jaya Diri’[Title/Abstract] OR ‘Sutami Reservoir’[Title/Abstract] OR ‘Southeast Asia’[Title/Abstract] OR ‘East Asia’[Title/Abstract] OR ‘Indo-pacific basin’[Title/Abstract] OR ‘ASEAN Countries’[Title/Abstract] OR ‘Indochina’[Title/Abstract] OR ‘Malay Archipelago’[Title/Abstract] OR ‘Maritime Southeast Asia’[Title/Abstract] OR ‘East Asia’[Title/Abstract] OR ‘Indo-Pacific Basin’[Title/Abstract] OR ‘Indo-Pacific Region’[Title/Abstract] OR ‘Asia-Pacific’[Title/Abstract] OR ‘South China Sea’[Title/Abstract] OR ‘Sulu Sea’[Title/Abstract] OR ‘Visayan sea’[Title/Abstract] OR ‘Sabah seas’[Title/Abstract] OR ‘Celebes Sea’[Title/Abstract] OR ‘Strait of Malacca’[Title/Abstract] OR ‘Java Sea’[Title/Abstract] OR ‘Banda Sea’[Title/Abstract] OR ‘Celebes Sea’[Title/Abstract] OR ‘Flores Sea’[Title/Abstract] OR ‘Arafura Sea’[Title/Abstract] OR ‘Timor Sea’[Title/Abstract] OR ‘Molucca Sea’[Title/Abstract] OR ‘Filipino’[Title/Abstract] OR ‘Filipina’[Title/Abstract] OR ‘Filipinos’[Title/Abstract] OR ‘Pinoy’[Title/Abstract] OR ‘Pinays’[Title/Abstract] OR ‘Indonesian’[Title/Abstract] OR ‘Indonesians’[Title/Abstract] OR ‘Malay’[Title/Abstract] OR ‘Malaysian’[Title/Abstract] OR ‘Malaysians’[Title/Abstract])

AND (((amnesic shellfish poisoning[MeSH Terms]) OR (ASP[Title/Abstract]) OR (azaspiracid shellfish poisoning[MeSH Terms]) OR (azp[Title/Abstract]) OR (ciguatera poisoning[MeSH Terms]) OR (ciguatera fish poisoning[MeSH Terms]) OR (CP[Title/Abstract]) OR (CTX[Title/Abstract]) OR (CTX-1[Title/Abstract]) OR (CTX-3B[Title/Abstract]) OR (CTX-3C[Title/Abstract]) OR (diarrhetic shellfish poisoning[MeSH Terms]) OR (dsp[Title/Abstract]) OR (neurotoxic shellfish poisoning[MeSH Terms]) OR (nsp[Title/Abstract]) OR (respiratory irritation[MeSH Terms]) OR (paralytic shellfish poisoning[MeSH Terms]) OR (psp[Title/Abstract]) OR (palytoxicosis[Title/Abstract]) OR (ostreopsis spp-algal syndrome[MeSH Terms]) OR (spp. algal syndrome[MeSH Terms]) OR (shellfish-implicated deaths[MeSH Terms]) OR (azaspiracid[Title/Abstract]) OR (azaspiracids[Title/Abstract]) OR (biotoxin[Title/Abstract]) OR (biotoxins[Title/Abstract]) OR (brevetoxin[Title/Abstract]) OR (brevetoxins[Title/Abstract]) OR (brevotoxin[Title/Abstract]) OR (brevotoxins[Title/Abstract]) OR (PbTx-2[Title/Abstract]) OR (PbTx-3[Title/Abstract]) OR (Aerosolized brevetoxin[MeSH Terms]) OR (aerosolized brevetoxins[MeSH Terms]) OR (ciguatoxin[MeSH Terms]) OR (ciguatoxins[MeSH Terms]) OR (domoic acid[MeSH Terms]) OR (isobaric PLTX[Title/Abstract]) OR (Neurotoxin[MeSH Terms]) OR (Neurotoxins[MeSH Terms]) OR (Okadaic acid and derivatives[MeSH Terms]) OR (dinophysis toxins[Title/Abstract]) OR (dynophisis toxin[Title/Abstract]) OR (dynophisistoxin[Title/Abstract]) OR (dynophisistoxins[Title/Abstract]) OR (ostreocin[Title/Abstract]) OR (ostreocins[Title/Abstract]) OR (ovatoxin[Title/Abstract]) OR (ovatoxins[Title/Abstract]) OR (palitoxin-like toxin[Title/Abstract]) OR (Palitoxin-like toxins[Title/Abstract]) OR (Palytoxin[Title/Abstract]) OR (Palytoxins[Title/Abstract]) OR (Palytoxin-analogues[Title/Abstract]) OR (Marine biotoxin[MeSH Terms]) OR (Marine biotoxins[MeSH Terms]) OR (Phycotoxins[MeSH Terms]) OR (PST[Title/Abstract]) OR (PSTs[Title/Abstract]) OR (Paralytic shellfish toxin[MeSH Terms]) OR (paralytic shellfish toxins[MeSH Terms]) OR (saxitoxin[MeSH Terms]) OR (saxitoxins[MeSH Terms]) OR (spirolide[Title/Abstract]) OR (spirolides[Title/Abstract]) OR (yessotoxin[Title/Abstract]) OR (yessotoxins[Title/Abstract])) OR ((‘Alexandrium affine’[Title/Abstract] OR ‘Alexandrium cohorticula’[Title/Abstract] OR ‘Amphidoma’[Title/Abstract] OR ‘Ceratium’[Title/Abstract] OR ‘Ceratium furca’[Title/Abstract] OR ‘Chaetoceros’[Title/Abstract] OR ‘Cochlodinium polykrikoides’[Title/Abstract] OR ‘Dinoflagellate’[Title/Abstract] OR ‘Dinoflagellates’ [Title/Abstract] OR ‘Dinophysis’[Title/Abstract] OR ‘Dinophysis sp’[Title/Abstract] OR ‘Karenia’[Title/Abstract] OR ‘Noctiluca’[Title/Abstract] OR ‘Prorocentrum sp’[Title/Abstract] OR ‘Protogonyaulax cohorticula’[Title/Abstract] OR ‘Pseudo-nitzschia’[Title/Abstract] OR ‘Pyrodinium’[Title/Abstract] OR ‘Skeletonema’[Title/Abstract] OR ‘Thalassiosira’[Title/Abstract] OR ‘Toxic phytoplankton’[Title/Abstract] OR ‘Trichodesmium’[Title/Abstract] OR ‘Clam’[Title/Abstract] OR ‘Clams’[Title/Abstract] OR ‘Aanadara spp.’[Title/Abstract] OR ‘Amusium pleuronecetes’[Title/Abstract] OR ‘Asian moon scallops’[Title/Abstract] OR ‘Arca spp.’[Title/Abstract] OR ‘Mussel’[Title/Abstract] OR ‘Mussels’[Title/Abstract] OR ‘Green mussels’[Title/Abstract] OR ‘Perna viridis’[Title/Abstract] OR ‘P. viridis’[Title/Abstract] OR ‘Perna viridis linnaeus’[Title/Abstract] OR ‘asian green mussel’[Title/Abstract] OR ‘green mussel’[Title/Abstract] OR ‘Tahong’[Title/Abstract] OR ‘Oyster’[Title/Abstract] OR ‘Oysters’[Title/Abstract] OR ‘Bivalve’[Title/Abstract] OR ‘Bivalves’[Title/Abstract] OR ‘Bivalve mollusc pinctada’[Title/Abstract] OR ‘Scallop’[Title/Abstract] OR ‘Scallops’[Title/Abstract] OR ‘Gastropod’[Title/Abstract] OR ‘Gastropod mollusc Turbo’[Title/Abstract] OR ‘Oliva vidua fulminans’[Title/Abstract] OR ‘Oliva’[Title/Abstract] OR ‘Olividae’[Title/Abstract] OR ‘Kalasiu’[Title/Abstract] OR ‘O.vidua fulminans’[Title/Abstract] OR ‘Crab’[Title/Abstract] OR ‘Crabs’[Title/Abstract] OR ‘Charybdis belcheri’[Title/Abstract] OR ‘C. belcheri’[Title/Abstract] OR ‘red crab’[Title/Abstract] OR ‘belcher’s crab’[Title/Abstract] OR ‘Lophozozymus pictor’[Title/Abstract] OR ‘Zosimus aeneus’[Title/Abstract] OR ‘Jania red alga’[Title/Abstract] OR ‘Demania alcalai’[Title/Abstract] OR ‘Demania reynaudii’[Title/Abstract] OR ‘D. alcalai’[Title/Abstract] OR ‘D. reynaudii’[Title/Abstract] OR ‘Barracuda’[Title/Abstract] OR ‘Rumpe’[Title/Abstract] OR ‘Blue marlin’[Title/Abstract] OR ‘Malasugi’[Title/Abstract] OR ‘Mackerel’[Title/Abstract] OR ‘Lupoy’[Title/Abstract] OR ‘Red emperor snapper’[Title/Abstract] OR ‘Lutjanus sabae’[Title/Abstract] OR ‘Crustacean’[Title/Abstract] OR ‘Crustacea’[Title/Abstract] OR ‘Mollusc’[Title/Abstract] OR ‘Brown tides’[Title/Abstract] OR ‘Cyanobacterial blooms’[Title/Abstract] OR ‘Dead zones’[Title/Abstract] OR ‘Discolored water’[Title/Abstract] OR ‘Discoloured water’[Title/Abstract] OR ‘HAB’[Title/Abstract] OR ‘HAB occurrences’[Title/Abstract] OR ‘HABs’[Title/Abstract] OR ‘Harmful Algal Bloom’[Title/Abstract] OR ‘Harmful Algal Blooms’[Title/Abstract] OR ‘Red tide’[Title/Abstract] OR ‘Seawater’[Title/Abstract] OR ‘Microalgae’[Title/Abstract] OR ‘Microalgal’[Title/Abstract] OR ‘Macroalgae’[Title/Abstract] OR ‘Macroalgal’[Title/Abstract] OR ‘marine poisoning’[Title/Abstract] OR ‘seafood intoxication’[Title/Abstract] OR ‘algal toxin’[Title/Abstract] OR ‘algal toxins’[Title/Abstract] OR ‘seafood safety’[Title/Abstract])))

Filter applied: Human

#### Search strings for Scopus and Web of Science

(‘Philippines’ OR ‘Luzon’ OR ‘Visayas’ OR ‘Mindanao’ OR ‘Palawan’ OR ‘Zambales’ OR ‘Pangasinan’ OR ‘Cavite’ OR ‘Pampanga’ OR ‘Bataan’ OR ‘Cambatutay’ OR ‘Metro Manila’ OR ‘Panay’ OR ‘Negros’ OR ‘Cebu’ OR ‘Bohol’ OR ‘Leyte’ OR ‘Samar’ OR ‘Masbate’ OR ‘Balete Bay’ OR ‘Balite Bay’ OR ‘Basilan’ OR ‘Bislig Bay’ OR ‘Casiguran’ OR ‘Aurora’ OR ‘Dauis’ OR ‘Davao’ OR ‘Davao Oriental’ OR ‘Davao Occidental’ OR ‘Hinatuan Bay’ OR ‘Lianga Bay’ OR ‘Litalit Bay’ OR ‘San Benito’ OR ‘Magueda Bay’ OR ‘Maguindanao’ OR ‘Santa Maria’ OR ‘Surigao’ OR ‘Honda Bay’ OR ‘Taytay’ OR ‘Malampaya Sound’ OR ‘Puerto Princesa’ OR ‘Taguines Lagoon’ OR ‘Camiguin Island’ OR ‘Canaco Bay’ OR ‘Bacoor Bay’ OR ‘Dumanguillas Bay’ OR ‘Murcielagos Bay’ OR ‘Zamboanga del Norte’ OR ‘Pilar’ OR ‘Sorsogon’ OR ‘Sual’ OR ‘Sapian Bay’ OR ‘Ivisan’ OR ‘Indonesia’ OR ‘Jakarta Bay’ OR ‘Kao Bay’ OR ‘Ambon Bay’ OR ‘Lampung Bay’ OR ‘Maninjau’ OR ‘Biak-Irian Jaya’ OR ‘Malaysia’ OR ‘Peninsular Malaysia’ OR ‘West Malaysia’ OR ‘Bachok’ OR ‘Johor’ OR ‘Kelantan’ OR ‘Kuantan’ OR ‘Malacca’ OR ‘Melaka’ OR ‘Pahang’ OR ‘Pantai Melawi’ OR ‘Sebatu’ OR ‘Sri Sebang’ OR ‘Sungai Geting’ OR ‘Teluk Bahang’ OR ‘Tumpat’ OR ‘East Malaysia’ OR ‘Malaysia Borneo’ OR ‘Sabah’ OR ‘Andus’ OR ‘Papar’ OR ‘Balambayan Island’ OR ‘Beaufort’ OR ‘Binnsuluk’ OR ‘Bongawan seashore’ OR ‘Gayana Resort’ OR ‘Inanam’ OR ‘Kota Kinabalu’ OR ‘Karambunai’ OR ‘Kg Kebagu’ OR ‘Kota Belud’ OR ‘Kota Marudu’ OR ‘Kuala Mengalong’ OR ‘Kuala menggatal’ OR ‘Kuala Penyu’ OR ‘Kuching’ OR ‘Likas Bay’ OR ‘Membakut’ OR ‘Miri’ OR ‘Numbak’ OR ‘Pelabuhan’ OR ‘Penampang’ OR ‘Penimbawan’ OR ‘Petagas’ OR ‘Pulau Gaya’ OR ‘Pulau Manukan’ OR ‘Pulau Sepangar’ OR ‘Rancangan Mawao’ OR ‘Sarawak’ OR ‘Sepanggar’ OR ‘Sepangar’ OR ‘Sg Mengkabong’ OR ‘Tanjung Aru’ OR ‘Tanjung Badak’ OR ‘Trayong’ OR ‘Tuaran’ OR ‘Serusup’ OR ‘Sipitang’ OR ‘Taman Jaya Diri’ OR ‘Sutami Reservoir’ OR ‘Southeast Asia’ OR ‘East Asia’ OR ‘Indo-pacific basin’ OR ‘ASEAN Countries’ OR ‘Indochina’ OR ‘Malay Archipelago’ OR ‘Maritime Southeast Asia’ OR ‘East Asia’ OR ‘Indo-Pacific Basin’ OR ‘Indo-Pacific Region’ OR ‘Asia-Pacific’ OR ‘South China Sea’ OR ‘Sulu Sea’ OR ‘Visayan sea’ OR ‘Sabah seas’ OR ‘Celebes Sea’ OR ‘Strait of Malacca’ OR ‘Java Sea’ OR ‘Banda Sea’ OR ‘Celebes Sea’ OR ‘Flores Sea’ OR ‘Arafura Sea’ OR ‘Timor Sea’ OR ‘Molucca Sea’ OR ‘Filipino’ OR ‘Filipina’ OR ‘Filipinos’ OR ‘Pinoy’ OR ‘Pinays’ OR ‘Indonesian’ OR ‘Indonesians’ OR ‘Malay’ OR ‘Malaysian’ OR ‘Malaysians’) AND (((amnesic shellfish poisoning) OR (ASP) OR (azaspiracid shellfish poisoning) OR (azp) OR (ciguatera poisoning) OR (ciguatera fish poisoning) OR (CP) OR (CTX) OR (CTX-1) OR (CTX-3B) OR (CTX-3C) OR (diarrhetic shellfish poisoning) OR (dsp) OR (neurotoxic shellfish poisoning) OR (nsp) OR (respiratory irritation) OR (paralytic shellfish poisoning) OR (psp) OR (palytoxicosis) OR (ostreopsis spp-algal syndrome) OR (spp. algal syndrome) OR (shellfish-implicated deaths) OR (azaspiracid) OR (azaspiracids) OR (biotoxin) OR (biotoxins) OR (brevetoxin) OR (brevetoxins) OR (brevotoxin) OR (brevotoxins) OR (PbTx-2) OR (PbTx-3) OR (Aerosolized brevetoxin) OR (aerosolized brevetoxins) OR (ciguatoxin) OR (ciguatoxins) OR (domoic acid) OR (isobaric PLTX) OR (Neurotoxin) OR (Neurotoxins) OR (Okadaic acid and derivatives) OR (dinophysis toxins) OR (dynophisis toxin) OR (dynophisistoxin) OR (dynophisistoxins) OR (ostreocin) OR (ostreocins) OR (ovatoxin) OR (ovatoxins) OR (palitoxin-like toxin) OR (Palitoxin-like toxins) OR (Palytoxin) OR (Palytoxins) OR (Palytoxin-analogues) OR (Marine biotoxin) OR (Marine biotoxins) OR (Phycotoxins) OR (PST) OR (PSTs) OR (Paralytic shellfish toxin) OR (paralytic shellfish toxins) OR (saxitoxin) OR (saxitoxins) OR (spirolide) OR (spirolides) OR (yessotoxin) OR (yessotoxins)) OR ((‘Alexandrium affine’ OR ‘Alexandrium cohorticula’ OR ‘Amphidoma’ OR ‘Ceratium’ OR ‘Ceratium furca’ OR ‘Chaetoceros’ OR ‘Cochlodinium polykrikoides’ OR ‘Dinoflagellate’ OR ‘Dinoflagellates’ OR ‘Dinophysis’ OR ‘Dinophysis sp’ OR ‘Karenia’ OR ‘Noctiluca’ OR ‘Prorocentrum sp’ OR ‘Protogonyaulax cohorticula’ OR ‘Pseudo-nitzschia’ OR ‘Pyrodinium’ OR ‘Skeletonema’ OR ‘Thalassiosira’ OR ‘Toxic phytoplankton’ OR ‘Trichodesmium’ OR ‘Clam’ OR ‘Clams’ OR ‘Aanadara spp.’ OR ‘Amusium pleuronecetes’ OR ‘Asian moon scallops’ OR ‘Arca spp.’ OR ‘Mussel’ OR ‘Mussels’ OR ‘Green mussels’ OR ‘Perna viridis’ OR ‘P. viridis’ OR ‘Perna viridis linnaeus’ OR ‘asian green mussel’ OR ‘green mussel’ OR ‘Tahong’ OR ‘Oyster’ OR ‘Oysters’ OR ‘Bivalve’ OR ‘Bivalves’ OR ‘Bivalve mollusc pinctada’ OR ‘Scallop’ OR ‘Scallops’ OR ‘Gastropod’ OR ‘Gastropod mollusc Turbo’ OR ‘Oliva vidua fulminans’ OR ‘Oliva’ OR ‘Olividae’ OR ‘Kalasiu’ OR ‘O.vidua fulminans’ OR ‘Crab’ OR ‘Crabs’ OR ‘Charybdis belcheri’ OR ‘C. belcheri’ OR ‘red crab’ OR ‘belcher’s crab’ OR ‘Lophozozymus pictor’ OR ‘Zosimus aeneus’ OR ‘Jania red alga’ OR ‘Demania alcalai’ OR ‘Demania reynaudii’ OR ‘D. alcalai’ OR ‘D. reynaudii’ OR ‘Barracuda’ OR ‘Rumpe’ OR ‘Blue marlin’ OR ‘Malasugi’ OR ‘Mackerel’ OR ‘Lupoy’ OR ‘Red emperor snapper’ OR ‘Lutjanus sabae’ OR ‘Crustacean’ OR ‘Crustacea’ OR ‘Mollusc’ OR ‘Brown tides’ OR ‘Cyanobacterial blooms’ OR ‘Dead zones’ OR ‘Discolored water’ OR ‘Discoloured water’ OR ‘HAB’ OR ‘HAB occurrences’ OR ‘HABs’ OR ‘Harmful Algal Bloom’ OR ‘Harmful Algal Blooms’ OR ‘Red tide’ OR ‘Seawater’ OR ‘Microalgae’ OR ‘Microalgal’ OR ‘Macroalgae’ OR ‘Macroalgal’ OR ‘marine poisoning’ OR ‘seafood intoxication’ OR ‘algal toxin’ OR ‘algal toxins’ OR ‘seafood safety’)))

Filter applied for Scopus: Human

Filters applied for Web of Science:

1. Geographical: Malaysia, Indonesia, Philippines
2. Research areas excluded: agriculture, geology, veterinary sciences, computer science, marine freshwater biology, engineering, zoology, energy fuels, physics, meteorology atmospheric sciences, palaeontology, geochemistry and geophysics, nuclear science technology, telecommunications, forestry, thermodynamics, construction building technology, entomology, polymer science, art, archaeology, imaging science photographic technology, mining mineral processing, urban studies, linguistics, biophysics, geography, arts humanities other topics, mechanics, metallurgy, architecture, astronomy and astrophysics, government law, communication, mathematical computational biology, history philosophy of science, religion, mycology, mathematics, physical geography, plant sciences, technology other topics, transportation, sport sciences

#### Complementary searches for Google Scholar

##### Philippines

###### Main intoxications

(‘paralytic shellfish poisoning’ OR ‘ciguatera poisoning’) Philippines (PIDSR OR DOH OR BFAR OR FETP OR surveillance OR advisory)

929 results

###### ASP/DSP/NSP cluster

(‘amnesic shellfish poisoning’ OR ‘diarrhetic shellfish poisoning’ OR ‘neurotoxic shellfish poisoning’) Philippines (DOH OR surveillance OR monitoring)

730 results

###### AZP Palytoxicosis/Ostreopsis

(‘azaspiracid shellfish poisoning’ OR ‘palytoxicosis’ OR ‘ostreopsis’) Philippines (surveillance OR DOH OR advisory OR monitoring)

479 results

###### Filipino language

(‘pagkalason sa pagkain-dagat’ OR ‘red tide’) Pilipinas DOH

Nine results

##### Malaysia

###### Main intoxications

(‘paralytic shellfish poisoning’ OR ‘ciguatera poisoning’) Malaysia (CDCIS OR MOH OR KKM OR Sabah OR surveillance)

829 results

###### ASP/DSP/NSP cluster

(‘amnesic shellfish poisoning’ OR ‘diarrhetic shellfish poisoning’ OR ‘neurotoxic shellfish poisoning’) Malaysia (surveillance OR MOH OR monitoring)

778 results

###### AZP + Palytoxicosis/Ostreopsis

(‘azaspiracid shellfish poisoning’ OR ‘palytoxicosis’ OR ‘ostreopsis’) Malaysia (surveillance OR Sabah OR monitoring)

537 results

###### Bahasa Malaysia

(‘keracunan kerang lumpuh’ OR ‘pasang merah’ OR ‘keracunan makanan laut’) Malaysia (KKM OR pengawasan)

10 results

### Appendix B

**Table B1.** Data extraction items

Record identification:
1. Record type (academic paper, conference procedures, reports, online databases, webpage, non-peer-reviewed reports, discussion papers, abstracts, commentaries, etc.)
2. Title
3. First author/Institution
4. Year of publication
5. Journal/Publisher
6. Country
7. Sample
8. Study design
9. Aim of the paper
10. Aim of the data collection (research, surveillance, monitoring, policy development, preparedness, etc.)
Intoxication characteristics:
11. Location
12. Intoxication
13. Exposure route (ingestion; airborne/inhalation; direct water contact)
14. Toxin
15. HAB type
Data source characteristics:
16. Type of provider (GPs, primary care centres, secondary care centres, local health authorities, insurances, research institutes, etc.)
17. Case finding approach (active vs. passive; active case finding involves proactive efforts to identify case on behalf of health workers (e.g., through door-to-door surveys, contact tracing and community outreach); passive case finding relies on existing health system infrastructure and cases are identified when individuals seek medical care)
18. Financing source (public vs. private initiatives – government grants, academic grants, NGOs and donors, international aid)
19. Frequency of collection [routine (daily, monthly) vs. occasional]
20. Monitoring period
21. Key actor in charge of the alert (designated individual vs. self-reporting)
22. Key actor in charge of collection
23. Collection system [surveys/questionnaires; disease registries; geographic information systems (GIS); public health surveillance systems and hotlines; electronic/medical health record; etc.]
24. Storage infrastructure [paper vs. digital (on-premise, cloud, centralised government servers, etc.)]
25. Data recipient (government, public authority, private sector, medical directorates, etc.)
26. Data accessibility (open vs. restricted access)
27. Disease coding method
28. The database is part of a broader monitoring network (*yes/no*)
29. The database is part of a temporary programme/intervention (*yes/no*)
30. The data are used for periodical elaboration of indicators (*yes/no*)
31. Other institutions responsible for surveillance/monitoring mentioned

### Appendix C

**Table C1.** Access constraints identified in the complementary consultation of national and supranational HAB-related health datasets

Dataset	Country/scope	Access limitation identified
FHSIS Annual Reports	Philippines	Published as aggregated PDF tables; PSP is the only HAB-related syndrome captured, with no disaggregation for ciguatera, ASP, NSP or palytoxicosis; coverage limited to public primary care facilities; mortality data not collected.
PIDSR Weekly Surveillance Reports	Philippines	PSP is formally a Category 2 notifiable disease but is not consistently reported in the publicly disseminated weekly bulletins, which prioritise vector-borne and vaccine-preventable diseases
DoH Freedom of Information (FOI) portal	Philippines	Request-based mechanism; case data honoured for individual years (e.g., 2020 PSP morbidity) with variable turnaround; not suitable for systematic longitudinal extraction
HAEDAT (IOC-UNESCO)	Supranational (21 Philippines, 10 Malaysia, 3 Indonesia events)	Openly accessible but structured around environmental bloom events rather than human illnesses; the IOC Asia regional network is still in a preparatory phase of contribution; illness counts, when present, appear as narrative rather than structured fields
CDCIS and Health Facts annual publications	Malaysia	HAB-related intoxications subsumed within the generic ‘food poisoning’ notifiable category (Prevention and Control of Infectious Disease Act 1988; published reports provide only aggregate national/state incidence; access to disaggregated data requires National Medical Research Register (NMRR) approval, generally restricted to in-country investigators
BPOM SIKERNAS and EWARS/SKDR	Indonesia	Data released only as descriptive PDF executive summaries (*Ringkasan Eksekutif Kajian Analisis Data Kasus Keracunan Obat dan Makanan*, 2022–2024); primary records not openly accessible; HAB toxins not disaggregated from broader seafood-related food poisoning
NHISD/*BPJS Kesehatan*	Indonesia	Accessible upon formal request; ICD-10 coding aggregates HAB-related intoxications under T61.x (‘toxic effect of seafood’), precluding HAB-specific extraction
ASEAN RASFF	Supranational (regional)	Closed network restricted to designated national food safety authorities; notifications concern contaminated food products in international commerce rather than individual case counts
INFOSAN (FAO/WHO)	Supranational (global)	Closed network restricted to designated national food safety authorities; same scope limitation as ASEAN RASFF
